# Impaired Learning of Social Compared to Monetary Rewards in Autism

**DOI:** 10.3389/fnins.2012.00143

**Published:** 2012-10-01

**Authors:** Alice Lin, Antonio Rangel, Ralph Adolphs

**Affiliations:** ^1^Computations and Neural Systems, California Institute of TechnologyPasadena, CA, USA; ^2^Division of Humanities and Social Sciences, California Institute of TechnologyPasadena, CA, USA

**Keywords:** social reward, monetary reward, autism

## Abstract

A leading hypothesis to explain the social dysfunction in people with autism spectrum disorders (ASD) is that they exhibit a deficit in reward processing and motivation specific to social stimuli. However, there have been few direct tests of this hypothesis to date. Here we used an instrumental reward learning task that contrasted learning with social rewards (pictures of positive and negative faces) against learning with monetary reward (winning and losing money). The two tasks were structurally identical except for the type of reward, permitting direct comparisons. We tested 10 high-functioning people with ASD (7M, 3F) and 10 healthy controls who were matched on gender, age, and education. We found no significant differences between the two groups in terms of overall ability behaviorally to discriminate positive from negative slot machines, reaction-times, and valence ratings, However, there was a specific impairment in the ASD group in learning to choose social rewards, compared to monetary rewards: they had a significantly lower cumulative number of choices of the most rewarding social slot machine, and had a significantly slower initial learning rate for the socially rewarding slot machine, compared to the controls. The findings show a deficit in reward learning in ASD that is greater for social rewards than for monetary rewards, and support the hypothesis of a disproportionate impairment in social reward processing in ASD.

## Introduction

Underlying the abnormal social behavior of autism may be social motivation deficits. One prominent theory, the social motivation hypothesis, attributes the social dysfunction to a deficit in reward processing and motivation specific to social stimuli (Dawson et al., 1998, 2002; Grelotti et al., 2002; Chevallier et al., 2012). In this framework, early-onset impairments in social motivation and attention set in motion developmental differences that ultimately deprive the child of adequate social learning experiences, which then leads to disruption in social skill and social cognition developments. The downstream effects of these disruptions result in the abnormal social behaviors of autism.

From the first year of life, individuals with autism spectrum disorders (ASD) show reduced interest toward social stimuli like faces, eye contact, and biological motion (Osterling and Dawson, 1994; Dawson et al., 1998; Klin et al., 2009). While non-ASD children have increased pupillary diameter to happy faces with direct gaze over averted gaze, children with ASD do not (Sepeta et al., 2012). These differences in individuals with ASD may be reflective of reduced reward motivation for social stimuli.

One key question about the motivation deficit is whether it is caused by a specific impairment in the neural reward processing of social stimuli, or if it reflects a more general deficit in learning stimulus-reward associations. A few neuroimaging studies have looked at social vs. non-social reward processing in ASD (Scott-Van Zeeland et al., 2010; Dichter et al., 2012), and a recent issue of the Journal of Neurodevelopmental Disorders has been devoted entirely to this topic (Dichter and Adolphs, 2012). Scott-Van Zeeland et al. (2010) reported that children with autism showed generally impaired implicit reward learning to both money and social stimuli, although the neural response to such stimuli measured with functional magnetic resonance imaging also showed a disproportionate abnormality for the social stimuli in particular. However, this study had several limitations. One limitation, common across many reward learning studies, was that only rewards and neutral outcomes were investigated; there was no condition for an aversive outcome. This raises the possibility that differences seen are due to attentional or arousal effects, which would be greater for rewards than neutral outcomes. Our present study (see below) included outcomes that were rewarding, neutral, or aversive. Another important feature of the study of Scott-Van Zeeland et al. (2010) is that the social outcomes were a person saying that the choice was right or wrong, thus confounding reward value with an error signal of whether the response made had been correct. These features of the task, perhaps together with the fact that the participants were all children rather than adults, resulted in a non-specific global deficit on task performance (for both social and monetary versions) in the ASD group in that study (Scott-Van Zeeland et al., 2010).

Another study (Dichter et al., 2012) found that the neural response to monetary reward learning was abnormal in people with ASD, but this abnormality disappeared during processing of interesting objects, possibly corresponding to the restricted interests common in the autism phenotype. However, this study had a limitation in that there was a large IQ difference between subject groups: the autism group had a nearly 20-point lower IQ than did the healthy controls, making it difficult to assign group differences to social reward processing rather than generally lower intellectual functioning. In our present study, we carefully matched each ASD participant one-for-one with each healthy participant, so that not only were our groups matched closely on IQ, but so were individual pairs of subjects. Finally, the study by Dichter et al. (2012) is somewhat different from ours in the processes that it investigated. It used an incentive delay task that essentially taps Pavlovian reward processing, whereas our task was instrumental. As in the Scott-Van Zeeland et al. (2010) study, an emphasis of the study by Dichter et al. (2012) was differences in regional brain activation from an fMRI task. By contrast our present study aimed to design a sensitive instrumental learning task that would show specific behavioral differences – building a platform for future fMRI studies.

In contrast to the above studies, a recent study looking at monetary rewards with event-related potentials (ERP) found typical reward outcome processing in the ASD group (McPartland et al., 2012). Similarly, Cascio et al. (2012) found that neural reward response measured with fMRI to food images showed very similar patterns of activation in both the ASD and control groups. Again, these studies put an emphasis on brain-derived measures rather than behavioral measures *per se*. The findings from these last two studies are broadly consistent with our findings in the present paper: non-social reward processing can be preserved in high-functioning people with ASD.

The above studies point to some inconsistencies in the literature in regard to reward processing in autism, leaving a key question unanswered: are reward processing deficits in ASD domain-general, or are they disproportionate for the social domain? Much of the discrepancy in the literature may result from factors such as inadequate matching of groups, from the various different tasks that have been used, from a failure to carefully match different types of rewards, and in particular from the complexities of obtaining neural measures (such as EEG and BOLD-fMRI) in clinical populations. In regard to the latter issue, it is particularly problematic to account for small differences in behavioral performance in the scanner, since these will influence the observed activations in complex ways. Our present study thus focused on a purely behavioral set of measures, emphasizing a carefully matched instrumental learning task that directly pitted monetary rewards and punishments against social rewards and punishments.

**Figure 1:**
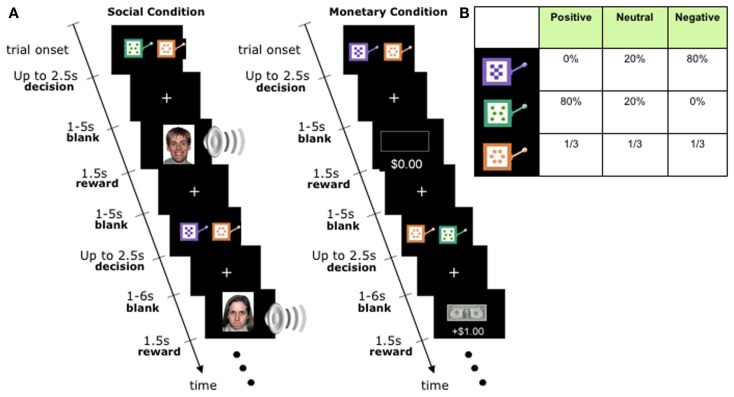
**Experimental Task**. **(A)** Timeline of the monetary and social reward trials. Choice trials paired a neutral slot machine with a valenced slot machine. Trials were identical except for the nature of the outcomes: monetary trials had a gain/loss of +$1, 0$, or −$1, whereas social trials revealed happy, neutral, or angry faces accompanied with sound effects of similar emotional valence. Specific slot machines were randomly assigned to specific reward outcomes at the start of the experiment for each subject, and distinct between monetary and social condition blocks. **(B)** Distribution of outcomes for each slot machine. First row: negative machine. Second row: positive machine. Bottom row: neutral machine. The same distribution was used in the monetary and social conditions. Actual appearance of the slot machines was randomly paired with a reward outcome distribution, and distinct between monetary and social condition blocks.

We used a task that provided structurally identical processing demands for social and non-social stimuli in the context of decision-making (Lin et al., 2012); see Figure 1. The task assessed the ability to learn to choose amongst options that were paired with different types of rewards: monetary in one version of the task, and social in a second version. By making the tasks otherwise structurally identical, differing only in the type of reward outcome obtained, we were able to provide a well-matched comparison to investigate the above questions.

The task (Figure 1) is of interest also because we have previously shown that it engages overlapping neural substrates in non-ASD populations (Lin et al., 2012). In line with a large literature on reward learning, the reward outcomes in the task activate the medial prefrontal cortex – and do so regardless of whether the reward is monetary or social. Similarly, decision values of the slot machines, and prediction errors that quantify the discrepancy between expected and obtained outcomes, also activate overlapping reward regions in the brain, regardless of whether the reward is monetary or social. This prior finding in healthy individuals using the same task helps to further constrain interpretations from the behavioral data in the present study (see Discussion).

We tested 10 high-functioning people with ASD (7M, 3F) and 10 healthy controls who were matched on gender, age, and education, on an instrumental reward learning task that contrasted learning with social rewards against learning with monetary rewards. In the social version of the task, outcomes were smiling, neutral, or angry faces accompanied by matching sound effects (happy, neutral, or angry voices). In the monetary version, outcomes were monetary gains or losses.

## Materials and Methods

### Participants

Twenty-seven subjects participated in the study (mean age = 22.4 years; range = 18–28). ASD subjects were matched one-to-one with healthy controls to ensure the best possible comparisons between groups. Seven ASD subjects were excluded from the analyses: four of these did not meet criterion on either the monetary or social task even though they appeared to understand the instructions; two did not understand the task instructions to begin with and could not do the task; and one did the task in valid fashion but failed to understand the subsequent rating instructions. Behavioral analyses reported are thus based on 20 subjects: 10 subjects with ASD (three female) and 10 age- and education-matched controls (three female; Table 1). All ASD participants met the Diagnostic and Statistical Manual of Mental Disorders, Revised 4th Edition diagnostic criteria for autism or Asperger syndrome and met the cutoff scores for autism or Asperger syndrome on the Autism Diagnostic Observation Schedule, Module 4 (Lord et al., 2000) and Autism Diagnostic Interview-Revised (Lord et al., 1994; Table 1). All participants had normal or corrected-to-normal vision, and had no history of psychiatric or neurological disease other than a diagnosis of autism spectrum disorder for the ASD participants. All healthy controls had a family history negative for an autism spectrum disorder. Participants gave informed consent to participate in this study under a protocol approved by the Caltech IRB.

**Table 1:** **Demographics and assessment background of participants**.

	*n* =	Gender	Age	FSIQ	Education (in years)	SRS
ASD	10	7M, 3F	28 (3.1)	113 (4.7)	15 (0.7)	76 (9.7)
			[18–45]	[93–133]	[10–18]	[24–113]
Matched controls	10	7M, 3F	27 (3.1)	114 (13.4)	15 (0.6)	56 (15.6)
			[17–44]	[104–123]	[12–18]	[41–72]

	ADI–A	ADI–B	ADI–C	ADI–D	ADOS–A	ADOS–B	ADOS–C	ADOS–D
ASD	20 (1.8)	17 (1.5)	6 (0.9)	3 (0.5)	4.9 (0.5)	10.2 (1.4)	1 (0.2)	1.4 (0.4)
	[12–28]	[11–24]	[2–12]	[0–5]	[3–7]	[4–17]	[0–2]	[0–3]

*FSIQ is full-scale IQ from the Wechsler Adults Intelligence Test (Wechsler, 1981). SRS is the social responsiveness scale (Constantino and Gruber, 2005), and ADI and ADOS are the Autism Diagnostic Interview (Lord et al., 1994) and Autism Diagnostic Observation Schedule (Lord et al., 2000). Means, SD, and range are provided on all subtests of the ADI and ADOS*.

### Task

Participants played two structurally identical versions of an instrumental learning task, one with monetary rewards, the second with social rewards (Figure 1). A trial began with the display of two visually distinctive slot machines, each associated with one of three outcome distributions: mean-positive, mean-negative, and mean-neutral.

All participants completed one social and one monetary block of 100 trials each; block order was randomized between participants. At the beginning of each trial, participants were shown a neutral slot machine paired with either the positive or negative slot machine (50/50 probability with randomized order). Participants chose one by pressing a left or right button. Up to 2.5 s were allowed for choice, followed by a uniformly blank screen displayed for 1–5 s (flat distribution), followed by the reward outcome displayed for 1.5 s, followed by an inter-trial interval of a uniformly blank screen displayed for 1–6 s (flat distribution). Participants were not told the reward probabilities associated with each slot machine and instead had to learn them by trial and error during the task (a process likely to be implicit rather than explicit, given the probabilistic nature of our task).

### Stimuli and rewards

The slot machines in both conditions were represented by cartoon images of actual slot machines that varied in color and pattern (Figure 1). The positive slot machine had mean-positive outcomes, the negative slot machine had mean-negative outcomes, and the neutral slot machine had mean-neutral outcomes. Please note that outcomes were probabilistically associated with the type of slot machine; thus, the neutral slot machine was in fact associated with a 1/3 probability of getting either a positive, negative, or neutral outcome (see Figure 1B for a breakdown of how each type of slot machine was associated with each type of outcome.)

In the social condition, reward outcomes were color photographs of unfamiliar faces from the NimStim collection (Tottenham et al., 2009) showing either an angry (negative outcome), neutral (neutral outcome), or happy (positive outcome) emotional expression, presented together with emotionally matched words played through headphones (normalized for volume and duration). Extensive prior piloting had demonstrated the behavioral efficacy of these stimuli in reward learning, as also evidenced by a prior study in an independent sample of healthy participants (see Lin et al., 2012). In the monetary condition, the positive outcome was a gain of one dollar (an image of a dollar bill), the negative condition was a loss of one dollar (image of a dollar bill crossed out), and the neutral condition involved no change in monetary payoff (image of an empty rectangle). Subjects were paid out the sum of their earnings at the end of the experiment.

### Face ratings and other post-task activities

At the end of the experiment, we asked subjects to rate the pleasantness of the social stimuli used in the imaging study. We were interested in whether the two groups experienced the stimuli similarly.

## Results

**Figure 2:**
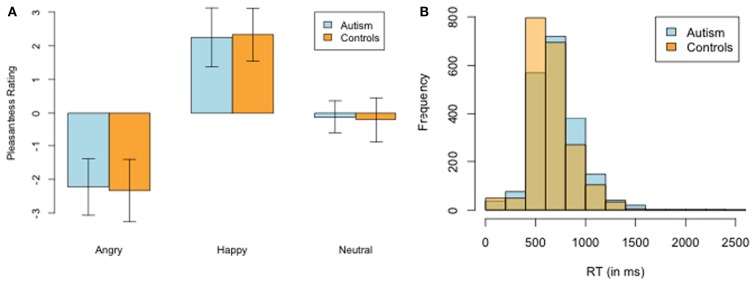
**Ratings of social stimuli**. **(A)** Pleasantness ratings of the happy, neutral, and angry social stimuli. There were no significant differences between groups on any of the categories. **(B)** Distribution of mean reaction-times between ASD and NT.

We compared a group of 10 high-functioning adults with ASD with 10 healthy controls matched on age, sex, and education (Table 1). Our first check was to confirm the two subject groups had similar subjective experiences of the face stimuli. Figure 2A plots the pleasantness ratings for angry, happy, or neutral social stimuli for each group. There were no statistically significant differences in any of the valence categories [Angry: ASD −2.23 vs. Controls −2.33; *t*(18) = 0.26, *p* = 0.80, Happy: ASD 2.25 vs. Controls 2.33; *t*(18) = −0.22, *p* = 0.83, Neutral: ASD −0.13 vs. Controls 0.22; *t*(17) = −0.87, *p* = 0.40] or in reaction-times [ASD Mean: 709 vs. Controls Mean: 655; *t*(18) = 1.60, *p* = 0.13; Figure 2B].

**Figure 3:**
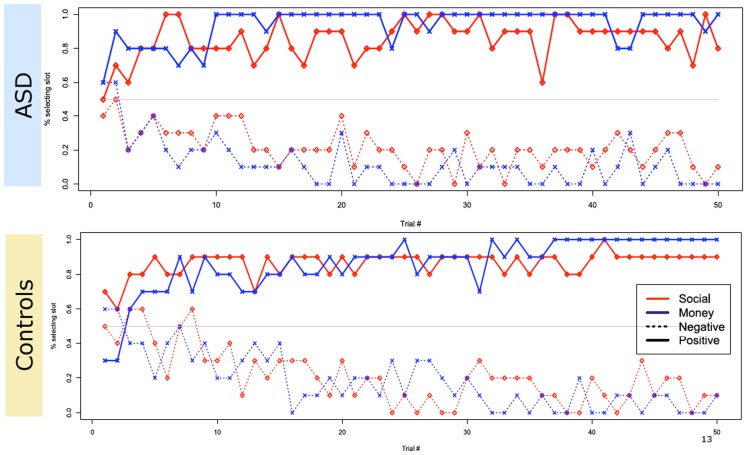
**Overall choice performance**. Plot of group subject choices across trials. Both groups reliably learned to select the slot machine associated with the highest probability of a positive-valenced outcome and avoid the slot machine associated with the highest probability of a negative valenced outcome, in both monetary and social conditions. The *y*-axis plots the percentage of total choices within each group that selected the optimal slot machine on a given trial.

Turning to the choice data from the main task, we found that both groups reliably learned to select the slot machine associated with the highest probability of a positive-valenced outcome for both social and non-social rewards, and to avoid the slot machine associated with the highest probability of a negatively valenced outcome (Figure 3).

**Figure 4:**
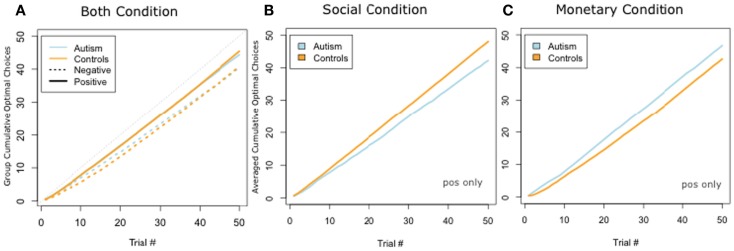
**Cumulative choices for positively valenced conditions**. **(A)** Plot of cumulative optimal responses across trials in social condition. **(B)** Plot of cumulative optimal responses across trials in the social condition. **(C)** Plot of cumulative optimal responses across trials in the monetary condition. We found a double dissociation, such that ASD participants cumulatively made a greater number of optimal choices in the monetary condition, whereas controls cumulatively made a greater number of optimal choices in the social condition.

We next plotted the cumulative number of optimal choices trial by trial. When collapsing over both social and monetary trials, ASD and Controls performed indistinguishably (Figure 4A). We ran a *t*-test comparing the slopes of the best fit-line of the two groups and found no difference [Positive: ASD 0.91 vs. Controls 0.92; *t*(17) = −0.38, *p* = 0.70; Negative: ASD 0.82 vs. Controls 0.84; *t*(13) = −0.41, *p* = 0.68]. However, when we separated out social and monetary trials, we found a double dissociation: ASD subjects were better than control subjects on the monetary condition but control subjects were better than ASD on the social condition (Figures 4B,C). Since we observed more rapid and more stable learning in the positive trials, we analyzed learning with two separate ANOVAs. In the first, we ran a 2 × 2 ANOVA of subject group (ASD or Controls) and reward condition (monetary and social) for the positive trials. We found no significant main effects of category or group (all *F*’s < 1, all *p*-values >0.6) but revealed a significant group by condition interaction effect [*F*(1,1) = 7.44, *p* < 0.01]. A *t*-test for differences of the slopes of the best fit-line for cumulative positive trials revealed slopes for the control group were significantly higher than the ASD group in the social condition [ASD: 0.86 vs. Controls: 0.97; *t*(10) = −2.35, *p* < 0.04]. Our second 2 × 2 ANOVA of subject group (ASD or Controls) and reward condition (monetary and social) with the negative trials did not reveal any significant main or interaction effects. We, therefore, focus only on the positive trials in the analysis that follows.

**Figure 5:**
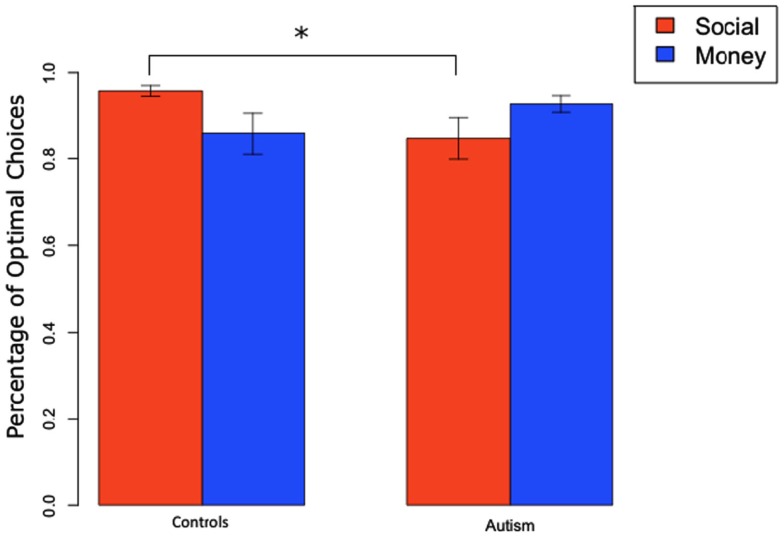
**Total percentage of optimal slot machine selection (mean and SEM) for positive trials in social and monetary condition**.

We found a similar result when we looked at total percentage of optimal slot machine selection at the end of the experiment (Figure 5). A 2 × 2 ANOVA of subject group (ASD or Controls) and condition (monetary and social), pooling positive and negative trials had no significant main effects of category or group (all *F*’s < 1, all *p*-values >0.4) but revealed a significant group by condition interaction effect [*F*(1,1) = 4.20, *p* < 0.05]. There was a significant difference between ASD and Controls on positive trials in the social condition [ASD: 0.85 vs. Controls: 0.96; *t*(10) = −2.27, *p* < 0.05] but not in any of the other conditions.

Lastly, a qualitative look at individual subject data suggested differing rates of learning. To capture this in a quantitative manner, we modeled each subject’s choice data with a probit regression, using the slope estimate of the probit regression as a metric for learning rate. We fit a probit regression through each subject’s raw data appended with 10 alternating left and right trials at the beginning. We padded the start to give the model enough learning trials since some subjects were able to identify the high value slot machine within one or two trials. Visual inspection confirmed this resulted in the best regression fit of the raw data.

We also checked whether the probit regression differentially modeled one group’s data better than the other’s by comparing Akaike Information Criteria (AIC) scores between the two subject groups and found no difference (all *p*-values > 0.05). After these checks, we felt confident that the slope estimate from each subject’s probit regression was an accurate reflection of learning rate.

**Figure 6:**
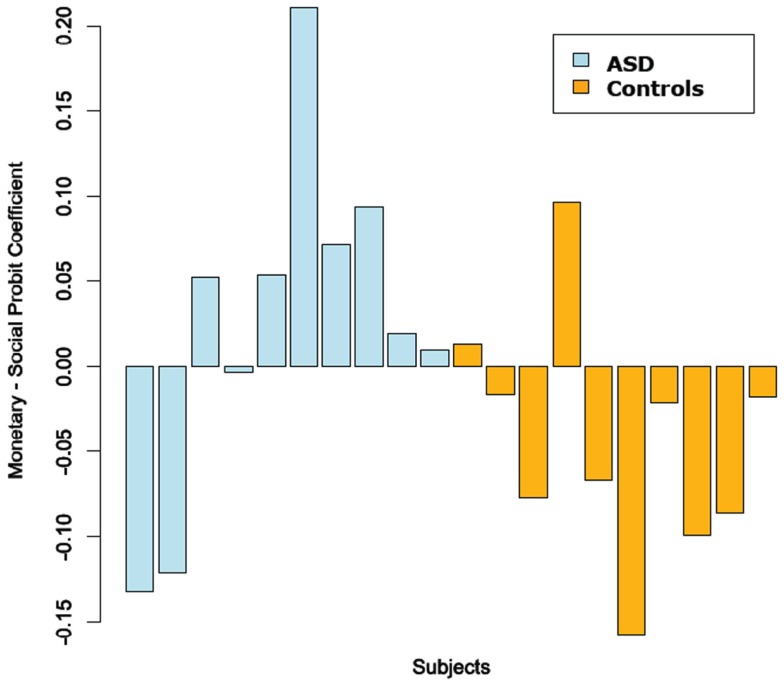
**Difference between monetary and social probit regression coefficients (positive trials only)**. We fit probit regressions to each subject’s choices on the positive trials in each condition. We then plotted the difference between the fitted monetary and social coefficient for each subject.

Figure 6 plots the difference between the probit slope coefficient for positive trials in the monetary and social condition for each subject. This showed an interesting group split. We quantified these findings with a Fischer’s exact test on the distribution of greater monetary vs. social probit slope coefficient in ASD and control subjects. We found there was a significant contingency between subject group (ASD vs. Controls) and whether he/she had a faster learning rate in the monetary over the social condition (*p* = 0.032). A greater proportion of participants in the ASD group, than participants in the control group, had learning rates that were faster for the monetary than the social condition. We also ran correlations between the ADOS, ADI, SRS, FSIQ, Age, and the social probit slope coefficients. None of these were statistically significant.

## Discussion

We investigated the hypothesis that people with autism spectrum disorder would show a disproportionate impairment in processing social rewards. Using a reward learning task that had identical structure, we administered two versions: one with social rewards, and one with monetary rewards. We compared two groups of participants: ASD and neurotypical controls, matched on age, gender, and IQ.

In terms of overall ability behaviorally to discriminate positive from negative slot machines in their choice behavior, reaction-times, and valence ratings, both groups performed remarkably similarly. Both groups learned to choose in favor of the slot machine associated with positive outcomes, and to choose so as to avoid the slot machine with negative outcomes; and both groups learned to do so for either the monetary or the social condition. The fact that both groups showed such similar choice behavior and gave essentially identical valence ratings to the social stimuli provides strong evidence that our ASD group did not have a basic perceptual impairment in recognizing the value of the social stimuli (the emotional faces we used), nor did they have a basic impairment in understanding the task or in showing motivated behavior to obtain rewards. The highly similar overall behaviors and ratings in the two well-matched groups provide a starting point for discovering more specific dissociations, to which we turn next.

When looking in more detail at the cumulative choices made, and at the rate at which participants learned to choose optimally, we found a disproportionate impairment in the ASD group in learning to choose social rewards, compared to monetary rewards. Over time, the ASD group selected significantly fewer of the most rewarding social slot machine compared to the monetary slot machine, and also had a significantly slower initial learning rate for the socially rewarding slot machine, compared to the monetary slot machine. This pattern of findings was particularly compelling because it went in the opposite direction to what we found in the controls. Whereas controls cumulatively made a greater number of optimal choices in the social than the monetary condition, the ASD group showed the converse pattern. Whereas controls generally learned faster in the social than the monetary condition, the ASD group again showed the converse pattern. These dissociations argue that the impairments in social reward processing found here in the ASD group cannot be attributed simply to an overall greater difficulty on the social than the monetary task. Rather, they appear to reflect a disproportionate impairment showing some domain-specificity for social rewards in people with autism. The findings demonstrate a subtle but specific behavioral insensitivity to social rewards in ASD, consistent with prior hypotheses.

With respect to the final level of performance, the ASD participants did not differ noticeably from the control group; they differed only in the rate of learning. Thus, the most apparent difference was early on in the task, where there is a significant difference in the slope of the learning curve in the ASD group compared to controls. In the aggregate, this results in a difference in the total cumulative number of correct trials. It leaves open the possibility that there may also be a processing deficit later in the task, but this could not be detected in our study due to inadequate power: basically, both groups are near ceiling later in the task, once they have learned. Thus, the impairment is only evident during learning in the present study; we are hopeful that future fMRI data could speak better to this point and possibly reveal processing differences in ASD not only during the initial learning phase, but also during later phases of the task.

An important limitation of the study is that our social stimuli are relatively artificial compared to real-life social encounters. It will be important to extent these investigations to stimuli that are more ecologically valid, and to learning situations more akin to everyday life. Nonetheless, the positive findings of the present study provide a particularly well controlled piece of evidence, and it may well be that the impairments in processing real-life social rewards are considerably more severe than what we found here. Another limitation to note is that our participants with autism were highly selected, limiting inference we can draw to the autism population in general. Specifically, all our participants with ASD were selected to be high-functioning, and furthermore they were selected to ensure that they produced valid task performances and could be reliably matched to healthy controls. This necessitated excluding a total of seven participants with ASD from a total sample of 17, yielding our final sample of 10. On the one hand, our approach ensures that the ASD group gave valid results and were best matched to the controls (thus erring on the side of being conservative in finding any group differences); on the other hand, this approach likely reduced sensitivity to find abnormalities, and would likely fail to find impairments that are present in the ASD population at large, especially if lower functioning individuals are included.

Future studies should extend these investigations to examining the underlying neural substrates. As our behavioral task has in fact been used in conjunction with fMRI (Lin et al., 2012), it lends itself well to model-based fMRI of reward learning and could be used to probe neural differences in ASD. Preliminary investigations along these lines are currently underway in our laboratory, but may require larger sample sizes to achieve the requisite power.

## Conflict of Interest Statement

The authors declare that the research was conducted in the absence of any commercial or financial relationships that could be construed as a potential conflict of interest.
